# Unmasking Potential Withdrawal Effects of Ashwagandha: A Case Report and Review

**DOI:** 10.7759/cureus.83577

**Published:** 2025-05-06

**Authors:** Andrew Holzman, Adam E Brown, Jackie Kelley, Douglas Rappaport, Wayne A Martini

**Affiliations:** 1 Emergency Medicine, Mayo Clinic Alix School of Medicine, Phoenix, USA; 2 Medicine, University of Arizona College of Medicine, Tucson, USA; 3 Emergency Medicine, Mayo Clinic, Phoenix, USA

**Keywords:** anxiety, ashwagandha, gaba modulation, herbal supplement withdrawal, insomnia

## Abstract

Ashwagandha (*Withania somnifera*) is a widely available herbal product often taken for stress-related concerns. While it is typically well-tolerated, individuals may occasionally experience side effects. Symptoms emerging after the discontinuation of ashwagandha have not been extensively documented. We report the case of a 20-year-old male who presented with tachycardia, insomnia, and symptoms of anxiety following abrupt discontinuation of ashwagandha extract (600 mg/day). His medical workup, including cardiac enzymes, electrocardiogram, complete blood count, liver function tests, and thyroid function tests, was unremarkable. His symptoms began shortly after cessation of ashwagandha, and no other clear medical or psychosocial stressors were identified. He was treated supportively with hydroxyzine for sleep and referred for outpatient psychiatric follow-up. This case may represent a withdrawal syndrome related to ashwagandha cessation, potentially mediated by its GABA-ergic activity. Although alternative explanations, such as recurrence of underlying anxiety, are possible, the timing and severity of symptoms after discontinuation suggest a withdrawal phenomenon. This case highlights the importance of assessing herbal supplement use in patients presenting with new-onset psychiatric or autonomic symptoms. Clinicians should be aware of the potential for withdrawal symptoms following ashwagandha cessation. A thorough history of supplement use should be included in the evaluation of patients presenting with anxiety, insomnia, or related complaints.

## Introduction

Ashwagandha (*Withania somnifera*), an herb that grows in Nepal, India, and the Middle East, is used predominantly for its calming effects [1]. Extracts are frequently compounded in pills or ground into powders for use in tea [2]. The herb has roots in traditional Indian medicine extending as far as 6,000 years, where it was used to promote longevity [3]. The dried root of Withania somnifera is listed in the Indian pharmacopeia as a drug [1].

In the United States, anxiety disorders and related conditions account for a significant number of emergency department (ED) visits annually. Between 2017 and 2019, approximately 4.6 million ED visits per year were attributed to anxiety, stress-related, or other nonpsychotic mental disorders, emphasizing the considerable burden of these conditions on emergency healthcare systems. It is important to note that these figures are based on data collected before the COVID-19 pandemic, which has been associated with increased prevalence of anxiety and other mental health conditions. As individuals increasingly turn to dietary supplements or more “natural” remedies like ashwagandha to manage anxiety and stress, it is critical for clinicians to understand both their potential benefits and risks. This includes recognizing possible adverse effects, such as withdrawal symptoms, that may complicate patient presentations.

Ashwagandha is sometimes classed as an “adaptogen,” a term used to describe plant-derived compounds believed to support return to homeostasis in the face of stressful stimuli [4]. This classification has been criticized for a potentially overbroad and unscientific nature [5]. Nevertheless, claims that ashwagandha extract has beneficial effects on anxiety and stress have found support in the literature, including limited evidence from randomized controlled trials [6].

Ashwagandha’s bioactivity has been attributed in part to its constituent withanolide compounds, including withanolide A, withanoside IV, and withaferin A [7]. The anxiolytic action of the withanolides may be mediated by GABA-ergic activity involving both direct modulation of the GABAA receptor and receptor upregulation [8].

Assessments of toxicity from ashwagandha have been largely favorable, with no teratogenicity and a potentially wide therapeutic window suggested in animal models [7]. Herbal supplements, despite being seen as “natural," may have adverse effects on the body [9]. A blinded and controlled safety study performed in India failed to find an association with negative outcomes for hematologic measurements and serum biomarkers, including liver and thyroid function [10]. Despite this, cases of hepatotoxicity have been reported. A case series of five individuals from Iceland and the United States described acute liver injuries involving cholestatic or mixed mechanisms [11]. In another case, the drug was associated with acute steatohepatitis, though in the context of alcohol use and polypharmacy with drugs and other supplements [12]. An acute liver injury indicated by elevated bilirubin and alanine transaminase in a 22-year-old with jaundice and itching was also reported [13]. One case of painless thyroiditis and thyrotoxicosis was described in a 47-year-old man two months after he began using ashwagandha [14].

We did not identify reports of addiction or withdrawal symptoms involving ashwagandha. A small amount of animal model evidence supports the use of the herbal extract in reducing morphine tolerance and alcohol dependence, potentially due to GABA modulatory effects [15].

Although we have primarily discussed ashwagandha’s use as an anxiolytic, patients may also use the herb for a variety of other purported indications, including a potential role in COVID-19 treatment and a number of endocrine effects. For example, ashwagandha extracts can be found in herbal “testosterone boosters” [16,17].

## Case presentation

A 20-year-old male, born in Saudi Arabia, attending college in the United States, was seen in the ED for anxiety and insomnia. The patient reported intermittent tachycardia, hypertension, lack of appetite, and sleeplessness for one week prior to seeking care. He denied major life stressors or suicidal ideation. He disclosed the use of marijuana through an electronic cigarette and 300 mg of ashwagandha extract twice daily. He ceased use of the ashwagandha two days prior to symptom onset.

Previously, the patient presented to an outside ED describing the same symptoms; a panic attack was suspected and treated with oral tablets of lorazepam. The patient returned to an outside clinic and the same outside ED on two other occasions for further opinions with the same diagnosis. Review of the medical record suggests ashwagandha use was not discussed during these encounters.

**Figure 1:**
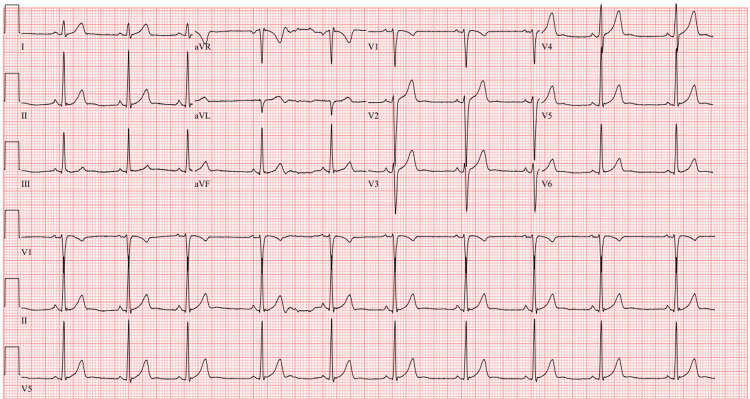
Electrocardiogram showing normal sinus rhythm with a heart rate of 61 beats per minute.

On evaluation, heart rate was mildly elevated to 89; blood pressure was 112/71. The temperature was 36.6 degrees Celsius, and the respiratory rate was 18, with a saturation of 95% on room air. There was a normal rate and regular rhythm on cardiac auscultation. The mental status exam was unremarkable with a normal mood and affect. D-dimer, troponin, and ECG (Figure 1) were obtained and were unremarkable. Labs, including hepatic function tests, CBC, and TSH, revealed a high hematocrit and erythrocyte count (Table 1).

**Table 1:** Laboratory values (H): Data are abnormally high.

Test name	Encounter result	Reference range and units
Hemoglobin	16.4	13.2-16.6 g/dL
Hematocrit	49.2 (H)	38.3-48.6 %
Erythrocytes	5.82 (H)	4.35-5.65 x10¹²/L
MCV	84.5	78.2-97.9 fL
RBC distribution width	12.5	11.8-14.5 %
Platelet count	296	135-317 x10⁹/L
Leukocytes	7.5	3.4-9.6 x10⁹/L
Neutrophils	5.23	1.56-6.45 x10⁹/L
Lymphocytes	1.59	0.95-3.07 x10⁹/L
Monocytes	0.52	0.26-0.81 x10⁹/L
Eosinophils	0.09	0.03-0.48 x10⁹/L
Basophils	0.03	0.01-0.08 x10⁹/L
D-dimer, P	319	≤500 ng/mL FEU
Sodium, S	138	135-145 mmol/L
Potassium, S	4.7	3.6-5.2 mmol/L
Chloride, S	102	98-107 mmol/L
Bicarbonate, S	24	22-29 mmol/L
Anion gap	12	7-15
BUN (blood urea nitrogen), S	19.2	8.0-24.0 mg/dL
Creatinine	0.92	0.74-1.35 mg/dL
Estimated GFR (eGFR)	>90	≥60 mL/min/BSA
Calcium, total, S	9.8	8.6-10.0 mg/dL
Glucose, S	91	70-140 mg/dL
Bilirubin, total, S	0.5	0.0-1.2 mg/dL
Alanine aminotransferase (ALT), S	22	7-55 U/L
Aspartate aminotransferase (AST), S	27	8-48 U/L
Alkaline phosphatase, S	93	40-129 U/L
Protein, total, S	7.6	6.3-7.9 g/dL
Albumin, S	4.8	3.5-5.0 g/dL
Troponin T, baseline, 5th gen	6	≤15 ng/L
TSH, sensitive	1.12	0.30-4.20 mIU/L

The patient’s presentation was attributed to cessation of ashwagandha use. Discontinuation and consideration of follow-up with a psychiatrist for therapy or initiation of a selective serotonin reuptake inhibitor (SSRI) were recommended. Hydroxyzine 10 mg tablet nightly as needed for sleep was prescribed. The patient was discharged from the emergency room. He returned two days later to obtain his prescription with no new symptoms.

## Discussion

**Figure 2:**
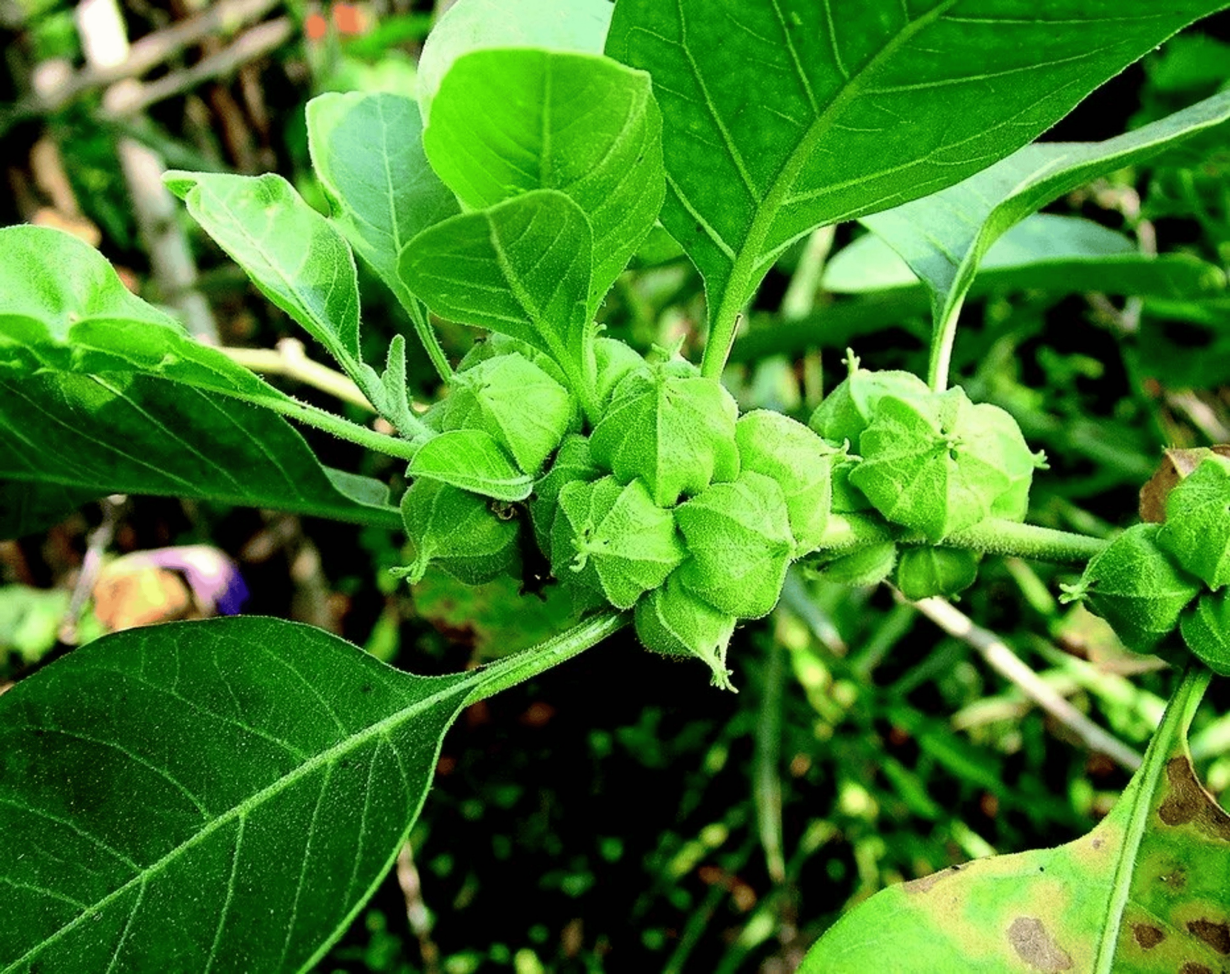
Ashwagandha (Withania somnifera) photo Photo By Vinayaraj, used under CC-BY-3.0 (cropped and compressed from the original)

This case represents an instance of withdrawal symptoms related to ashwagandha (Figure 2) use for its anxiolytic properties. Withdrawal syndromes involving alcohol and benzodiazepine cessation are well described and are understood to be caused by chronic tolerance of high GABAergic tone. Following cessation of the relevant agonist, central nervous system excitatory response to glutaminergic tone produces symptoms including tachycardia, hypervigilance, and others [18]. These mechanisms may relate to the addictive potential of GABA agonists [19].

In addition to the withdrawal mechanism, it is possible that this patient’s presentation is attributable to the underlying symptoms he sought to treat with herbal supplementation. We are unable to differentiate with precision the degree to which this contributed to this case, although the patient’s repeated presentation to the ED suggests his symptoms were new and acutely worse than what he had previously experienced. We note that our patient complained in particular of trouble sleeping; ashwagandha’s role as a GABA agonist may be particularly helpful in aiding sleep [20]. The patient’s intake of ashwagandha at 600 mg/day compares reasonably to doses described in the literature, which vary widely, with one review finding positive dose-dependent effects to 12,000 mg for anxiety and 600 mg for chronic stress [6].

We note that it appears this patient’s supplement use was not explored during his previous repeated presentations for medical evaluation. In the United States, approximately 23,000 annual visits to the ED each year may be attributable to the use of dietary and herbal supplements [21]. Evaluation of herbal and dietary supplementation is an important part of ED practice. Withdrawal and addictive potential have been prominently described in the case of another herbal supplement, a Mu opioid receptor agonist known as kratom [22].

## Conclusions

As the use of over-the-counter herbal supplements continues to grow, clinicians must remain vigilant in recognizing both their therapeutic potential and associated adverse effects, including those that may arise after discontinuation. This case adds to the limited literature suggesting that ashwagandha, while generally regarded as safe, may cause clinically significant withdrawal symptoms in some individuals, potentially through its interaction with GABAergic pathways. The constellation of symptoms observed in this patient, including tachycardia, anxiety, and insomnia, mirrored withdrawal phenomena seen with other central nervous system depressants, supporting the possibility of a physiological dependence or homeostatic disruption following cessation.

While the exact mechanism underlying this response remains to be elucidated, the temporal association, absence of other identifiable causes, and resolution with supportive care all underscore the importance of further exploration into ashwagandha's pharmacodynamics and potential for withdrawal. In the absence of established clinical guidelines, providers should counsel patients about the possible risks of abrupt discontinuation, especially in those taking high doses or using ashwagandha chronically.

This case serves as a timely reminder to include detailed questions about supplement use during patient evaluations, particularly in younger individuals presenting with anxiety-spectrum symptoms or autonomic instability. Future studies and pharmacovigilance efforts are needed to characterize the prevalence, risk factors, and underlying mechanisms of ashwagandha withdrawal. Until then, this case advocates for a cautious and informed approach to both the initiation and discontinuation of herbal products in clinical practice.
